# Structure and Development of the Legume-Rhizobial Symbiotic Interface in Infection Threads

**DOI:** 10.3390/cells10051050

**Published:** 2021-04-29

**Authors:** Anna V. Tsyganova, Nicholas J. Brewin, Viktor E. Tsyganov

**Affiliations:** 1 Laboratory of Molecular and Cellular Biology, All-Russia Research Institute for Agricultural Microbiology, 196608 Saint Petersburg, Russia; vetsyganov@arriam.ru; 2 Formerly Emeritus Fellow, John Innes Centre, Norwich NR4 7UH, UK; nick.brewin@gmail.com

**Keywords:** arabinogalactan protein, cell wall, extensin, infection thread, legume, pectin, *Rhizobium*, symbiosis

## Abstract

The intracellular infection thread initiated in a root hair cell is a unique structure associated with *Rhizobium*-legume symbiosis. It is characterized by inverted tip growth of the plant cell wall, resulting in a tunnel that allows invasion of host cells by bacteria during the formation of the nitrogen-fixing root nodule. Regulation of the plant-microbial interface is essential for infection thread growth. This involves targeted deposition of the cell wall and extracellular matrix and tight control of cell wall remodeling. This review describes the potential role of different actors such as transcription factors, receptors, and enzymes in the rearrangement of the plant-microbial interface and control of polar infection thread growth. It also focuses on the composition of the main polymers of the infection thread wall and matrix and the participation of reactive oxygen species (ROS) in the development of the infection thread. Mutant analysis has helped to gain insight into the development of host defense reactions. The available data raise many new questions about the structure, function, and development of infection threads.

## 1. Introduction

During the course of evolution, plants have exploited certain properties of microorganisms to expand their functional capabilities. Legumes and actinorhizal plants belong to the nitrogen-fixing clade Fabids or Eurosid I, collectively termed the FaFaCuRo [1,2]. These plants have acquired the ability to develop endosymbiotic relationships with various proteobacteria, collectively called rhizobia, and with actinobacteria from the genus *Frankia* [3,4,5]. In these symbioses, prokaryotes fix nitrogen derived from the air, which is provided to host plants in exchange for carbon sources derived from photosynthesis. One of the features associated with the symbiotic interaction is the formation of specialized organs called root nodules which provide a suitable microenvironment for bacterial nitrogenase activity. Nodule development requires the synthesis and recognition of signal molecules and structural components that are produced by both bacterial and plant partners [6].

To penetrate plant roots, bacteria follow different routes and use a variety of entry mechanisms that are determined by the host plant [7]. Probably the most primitive mechanism is intercellular root penetration, which is present in at least 25% of all legume genera [7,8]. This type of infection is morphologically simpler than the formation of intracellular infection threads occurring through the root hair cell, a process that apparently arose later in evolution. In the case of intercellular invasion, rhizobia can penetrate into the host tissue by several different routes: through the middle lamellae between adjacent root hair cells; through wounds arising during lateral root appearance (‘crack entry’); and directly between cells of the intact epidermis [7,8,9,10,11]. In this case, the subsequent colonization of the nodule primordium occurs as a result of intercellular infection, which is accompanied by the formation of tubular intercellular structures that resemble intracellular infection threads but lack the property of polar cell wall growth [12]. In some species of legumes with an intercellular infection, the bacteria within infection threads develop the capacity for nitrogen fixation. Such structures are called fixation threads [10].

The most well-characterized process of infection involves intracellular infection threads and occurs through root hair cells. About 75% of all legumes studied form infection threads in this way [7,13]. In 1887, Ward [14] described small ‘hyphae’ (which we now describe as infection threads) passing through the lumen of cells and through their walls. This was observed during the infection of clover, pea, vetch, beans, and other legumes. Early investigators believed that infection threads represented bacteria trapped in mucous threads. However, McCoy [15] observed that the mucous thread was encased in a sheath having the same general composition (cellulose, hemicellulose, and possibly some pectin) as the walls of young plant cells [16]. It is now considered that rhizobia penetrate the root hair cells through tubular structures bounded by plant cell wall material. Infection threads serve as a channel for the colonization of bacterial cells that grow and divide in their lumen, which is filled with a plant-derived extracellular matrix [17].

Infection threads are unique cell wall invaginations of plant origin. In many cases, they are able to traverse host cells, apparently fusing with the wall on the opposite side of the cell, thereby releasing bacteria into the intercellular matrix. Apical growth of an intracellular infection thread resembles tip growth of root hairs and pollen tubes [18], except that the orientation is ‘inside-out.’ This means that tubular growth proceeds into the cell [19,20,21]. In summary, in different legumes, infection threads can either grow through the intercellular space (intercellular infection threads) or through cells (intracellular or transcellular infection threads). The present review is concerned with the structure and development of intercellular and transcellular infection threads, which are characterized principally by the remodeling of plant cell wall growth and differentiation.

An infection thread is not just an ingrowth of a plant cell wall but a complex symbiotic structure [22,23]. It includes components of plant origin (cell wall polysaccharides, extracellular matrix glycoproteins such as arabinogalactan proteins (AGP), hydroxyproline-rich glycoproteins (HRGP), glycine-rich glycoproteins, extensins and others, as well as, various enzymes, receptors, and structural proteins) and also components of bacterial origin (both polysaccharides and proteins). During the infection of host tissue, the physical interaction between the bacterial and plant cell surfaces becomes progressively more intimate [24,25]. At each stage, the symbiotic interface must adapt thus that bacteria can exist in the new environment and avoid the development of defense reactions by the host plant [17,22].

Underpinning the infection process is a network of species-specific plant-microbial signal exchanges that involve lipochito-oligosaccharide Nod factors. These interactions have been extensively described [6,26,27,28]. Using model legumes for genetics and genomics, an ever-increasing range of plant genes has been identified that apparently contribute to the infection process. These genes encode transcription factors, LysM receptor kinases, E3 ubiquitin ligases, the Suppressor of cAMP receptor defect/WASP family verpolin homologous protein (SCAR/WAVE) actin regulatory complex, nitrate transporters, remorins, flotillins, proteins involved in biogenesis and membrane movement, and numerous other components [29,30,31]. Very little is known about the direct relevance of these components to the structure and development of infection threads, the dynamics of the polysaccharides of the of cell walls, and the extracellular matrix [17].

In this review, we will explore the sequential development of the symbiotic interface, which involves the remodeling of the cell wall and extracellular matrix during the growth of infection threads. This process stretches from the early stages of tissue invasion in root hairs through to the stage when biological nitrogen fixation develops within the host cells of mature nodules.

## 2. Molecular Dialogue

The study of chemical signaling in the interaction of plants and microorganisms has shown the existence of a precise molecular dialogue before and during direct contact between plant and bacterial cells [28,30,32]. Nod factors of rhizobia bind in the cell wall of root hairs [33] and cause a number of physiological and morphological plant reactions, such as depolarization of the host cell membrane [34], production of reactive oxygen species (ROS) [35], spiking of intracellular Ca^2+^ [36,37], and reorganization of actin microfilaments and endoplasmic microtubules in the tip of the root hair [38,39,40,41]. The first morphological changes observed under the action of Nod factors on legumes are deformations and curling of root hairs.

## 3. Attachment

### 3.1. Attachment of Rhizobia

Although deformation of root hairs can occur in response to Nod factor, intense curling is only possible after attachment of rhizobia [42,43]. As a prelude to infection, rhizobial cells accumulate on the surface of legume roots, forming a biofilm [44,45]. The increased population of bacterial cells in the biofilm amplifies the bacterial signal, thereby increasing the response from the host plant. The first phase of attachment is through weak, reversible, and nonspecific binding. The second stage is associated with the synthesis of bacterial cellulose microfibrils, which further strengthens the binding of bacteria to the roots. Other factors, such as pH, Ca^2+^, and Mg^2+^ concentrations, specific growth conditions, and root pretreatment, can also affect the attachment of rhizobia to the root surface [46].

It had previously been suggested that the binding of plant lectins to bacterial cell surface polysaccharides was part of the mechanism that determines the specificity between rhizobia and their legume hosts [47]. However, it is now generally accepted that this ‘lectin hypothesis’ was incorrect. The main determinants of host specificity are Nod factors, not surface polysaccharides [43,48]. Attachment of rhizobial cells to roots is considered to be a non-specific process and independent of symbiotic properties [42,49].

*Rhizobium leguminosarum* bv. *viciae* can use at least two mechanisms for attachment to root hairs of *Pisum sativum* L. and *Vicia sativa* L. One of them involves plant lectin, while the other is mediated by bacterial rhicadhesin [50]. In an acidic environment, lectin on the surface of root hairs binds to the polarized surface polysaccharide glucomannan produced by *R. leguminosarum* [50,51]. Thus, plant lectins can influence the extent of nodulation in legumes [52]. However, a complicating factor is that lectins can also modulate plant defense responses. During pathogenic interactions, lectins may become associated with receptor proteins in membrane microdomains. This strengthens the host defense response, but this effect is apparently weakened in a mutualistic symbiosis [53,54]. Nod factor could play a role in suppressing the defense response through either a direct or indirect interaction with the Lectin receptor kinase (LecRK) [55,56].

Rhicadhesin, a Ca^2+^-binding protein of *R. leguminosarum*, has been shown to increase adherence to root hairs under alkaline conditions [43,57]. A similar protein has been identified in *Bradyrhizobium* spp. [58]. Using a phage display library from *R. leguminosarum*, several Rhizobial attachment proteins (RAPs) were identified [59,60]. These proteins are secreted through the inner and outer membranes via a Type I secretion system, encoded by the *prsD* and *prsE* genes [61]. Calcium-binding adherence proteins (cadherins) are also secreted through the PrsDE system [62].

Legume annexins can also play a role during the early stages of infection [63]. The symbiotic annexins MtANN1 and MtANN2 have been shown to be associated with individual symbiotic events [64], in particular, with Ca^2+^ spiking [63,65]. Another possible participant in rhizobial attachment is the arabinogalactan protein (AGP). This new mode of binding may be important for the growth of rhizobia on the roots of both legumes and non-legumes [66].

### 3.2. Curling of Root Hairs

Rhizobial attachment is closely associated with Nod factor-induced deformation of the root hairs, which undergoes curling through 360-degrees [22,67]. Curling requires living bacterial cells [68]. It disrupts the normal pattern of polar growth of the root hair tip, resulting in the trapping of bacteria within the curl, called the ’shepherd’s crook.’ Here, the bacteria multiply to form a microcolony [67,69].

The actively growing tip of the root hair cell has a characteristic polarized organization [70,71]. It is enveloped by the cristalline layer of the cell wall, behind which is a dense cytoplasm filled mainly with secretory vesicles that are located along the actin microfilaments or microtubules [72]. The nucleus follows the advancing tip of the root hair at a fixed distance [72,73].

Successful invasion involves a reorientation of plant cell wall growth to allow initiation of an infection thread by inward growth into the root hair cell. After the Nod factor is recognized by receptor kinases, Ca^2+^ spiking in root hairs initiates the downstream signaling events [30,32]. Calcium spiking is also involved in the initiation of the tip growth of root hair cells and pollen tubes [18,71]. In root hairs, the nuclear envelope and the endoplasmic reticulum associated with the nucleus are potential internal stores of Ca^2+^ to be released during Ca^2+^ spiking [74]. The creation of artificial Ca^2+^ gradients in the root hair using ultraviolet-activated ionophores indicates that the establishment of the Ca^2+^ gradient is sufficient to initiate root hair growth. In this case, a temporary shift occurs in the direction of growth from the tip to the site of the induced gradient [75].

Curling of root hairs and a change in the direction of growth are correlated with and probably caused by changes in the plant cytoskeleton [76]. Actin depolymerization and reorganization of both endoplasmic and cortical microtubules are some of the earliest effects observed in root hairs following exposure to the Nod factors [38,40,41]. Following root hair curling, the tips of the root hairs swell, the number of subapical fine bundles of actin filaments (FB-actin) increases [39], and the microtubular cytoskeleton is re-formed [40,41]. Recently, it was demonstrated that microtubule reorganization during rhizobial infection in *Medicago truncatula* Gaertn. is regulated by Developmentally regulated plasma membrane polypeptide (DREPP), a member of the DREPP/PCaP family of microtubule-binding proteins [77].

Based on the phenotypes of legume mutants defective at successive stages of nodule development, substantial progress has been made in elucidating the mechanisms controlling the processes of infection [29,31,78,79]. Inhibition of the *M. truncatula* Phosphatidylinositol 3 kinase (*MtPI3K*) gene (regulating vesicle trafficking and the oxidative burst) led to decreased root hair curling and infection thread initiation. This indicated an important role for the vesicle trafficking system and for ROS in the initial steps of rhizobial colonization [80]. The curling of root hairs is also mediated by the Rho family of small GTPases (ROP). In *M. truncatula*, ROP10 is localized on the plasma membrane at the tips of root hairs. Interactions between ROP10 and Nod factor receptors are required for root hair deformations and curling during rhizobial infection [81].

## 4. Invasion of Host Cells

### 4.1. Initiation of the Infection Thread

As a result of root hair curling, rhizobia are trapped in a confined space [82]. Continued growth and division lead to the formation of a microcolony, which develops within the infection chamber (pocket). Rhizobia inhabiting the infection thread are the descendants of only a few founder cells derived from the initial infection event [83]. Gradual enlargement of the microcolony is accompanied by a rearrangement of the infection chamber. The entrapped bacteria generate a high local concentration of Nod factor, which may stimulate the initiation of an infection thread [84,85]. Thus, localized production of Nod factor within the infection pocket may act as a morphogenic organizing center, providing positional information for cell wall remodeling through reorientation of the underlying plant cytoskeleton.

During the modification of the infection chamber and initiation of the infection thread, many different proteins are involved. For example, upon inoculation of *M. truncatula* with *Sinorhizobium meliloti*, rearrangement of the infection chamber is accompanied by accumulation of a marker for exocytosis, the Vesicle-associated membrane protein 721e (MtVAMP721e) [20,86]. Intensive synthesis of the infection-associated secreted protein Early nodulin 11 (MtENOD11) begins around the rhizobia trapped in the chamber. This may increase the cell wall plasticity required to reduce turgor and radial expansion, followed by the initiation of inward polar growth of the infection thread [20]. ENOD11 is a proline-rich protein that contains a reduced amount of tyrosine, which probably limits its cross-linking with other cell wall components.

Other plant components involved in the maturation of the infection chamber and the initiation of the infection thread were identified in different legumes: E3 ubiquitin ligase Cerberus [87], the SCAR/WAVE complex [76], two flotillins [88], vapirin [89], a nonspecific lipid transfer protein N5 protein (MtN5) [90], Lack of symbiont accommodation (LAN), acting as a subunit of the mediator complex [91], transcription factors CYCLOPS/IPD3 (Interacting protein with DMI3) [92], NSP1 (Nodulation signaling pathway 1) [93], NSP2 [94], ERF required for nodulation (ERN) [95], Nodule inception (NIN) [96], CCAAT-box-binding Nuclear factor YA1 (NF-YA1) and NF-YA2 [97]. Growth of the infection thread in the root hair requires the movement of nuclei and recently the involvement of Linker of nucleoskeleton and cytoskeleton (LINC) complexes was demonstrated [98].

Three different explanations have been proposed for structural changes leading to bacterial penetration into the root hair cell as part of an incipient infection thread. First, Nutman [99] proposed invagination of the root hair cell wall, in which the growth direction of the plant cell wall changes at a localized point so that it grows back into the root hair, forming a tubular infection thread. Second, Ljunggren and Fåhraeus [100] proposed the ‘polygalacturonase’ hypothesis, according to which rhizobial exopolysaccharide increases the activity of plant polygalacturonase, and an individual bacterial cell dissolves cell wall pectins and subsequently penetrates through it without obvious structural damage. The infection thread is formed as an encapsulation response upon contact of the rhizobia with the plasmalemma. Finally, Dart and Mercer [101] proposed the penetration of small coccoid forms of rhizobia through cracks in cellulose microfibrils.

Currently, it is thought that the initiation occurs by remodeling of the cell wall and ingrowth of the infection thread using some form of inverted tip growth mechanism [17,83]. Structural disorganization of the cell wall of root hairs has been demonstrated at the site of infection thread initiation and is associated with direct contact between rhizobial cells and the plasma membrane [102,103,104]. Subsequent initiation of an infection thread wall probably involves the participation of bacterial and plant enzymes that modify cell wall polysaccharides. Rhizobia have enzymes that can degrade cellulose and other polysaccharides of the plant cell wall [54]. In addition, to initiate an altered growth process, rhizobia can induce the production of plant polygalacturonases (PGs) and pectin methylesterases (PMEs) [105,106,107]. Plants are also able to modify the composition of their cell walls [108,109]. In response to *S. meliloti,* the gene for *M. sativa* polygalacturonase (*MsPG3*) is induced [110,111]. The *Nodulation pectate lyase* gene (*LjNPL*) was identified in *Lotus japonicus* (Regel) K. Larsen, [112], and expansins are also involved in the infection process [113]. It should be emphasized that the wall of the infection thread is topologically continuous with the host cell wall and encapsulates the rhizobia [102,103,104]. There is no direct penetration through the plasmalemma: Bacteria always remain in the apoplastic space of root hair cells [102].

Both actin [80] and microtubular [114] cytoskeletons are involved in the initiation of infection threads. When the actin cytoskeleton is disturbed, and in particular when the fine F-actin is disorganized, it results in defective growth of infection threads, pollen tubes and root hairs. This phenotype was observed in the mutant *crinkle* of *L. japonicus* [115]. Mutants in *L. japonicus Actin-related protein component 1* (*LjARPC1*) gene, which encodes the Actin-related protein 2/3 (APR2/3) subunit of the complex that controls the nucleation of Y-shaped branched actin microfilaments, formed a reduced number of microcolonies [116]. *L. japonicus* mutants *121F-specific p53 inducible RNA* (*Ljpir1*) and *nck-associated protein 1* (*Ljnap1*) [76], as well as the *M. truncatula required for infection thread* (*Mtrit–1*) mutant (ortholog *Ljnap1*) [117] were characterized by a similar phenotype (disorganization of the actin cytoskeleton, no reorganization of F–actin in response to inoculation, a decrease in the number of microcolonies in curled root hairs) [76,116]. In addition, Actin reorganization is regulated by the activation of the ROP GTPase family [118], inositol phospholipids [119], and actin depolymerization factor (PvADFE) in the *Phaseolus vulgaris* L.-rhizobia symbiosis [120].

ROS and NO also play an important role in the initiation and growth of infection threads [121]. Nod factors can activate the first wave of ROS production, which is involved in nodule development, and they also inhibit the second wave, which is involved in defense responses [122,123]. The first wave modulates the expression of plant genes and/or the redox status of proteins involved in root hair deformation [123], infection thread progression, and nodule formation [124,125,126]. For the second wave, suppression of immune responses (ROS production, and accumulation of salicylic acid) was observed in the roots of *M. truncatula* and *M. sativa* upon addition of Nod factors [122,127]. In addition to ROS effects, there is an initial release of NO at the early stage of symbiotic interaction. This induces the expression of non-symbiotic hemoglobin (ns-Hb), which, in turn, traps NO and reduces the plant defense response [128,129,130].

Class III peroxidases (Prx-III) are considered as potential sources of enzymatic ROS. Examples include *Rhizobium*-induced peroxidases (Rip1-10) [131,132] and NADPH oxidases, also called Respiratory burst oxidase homologues (Rbohs) [123,125,126].

### 4.2. The Nodule Primordium and Nodule Meristem

Simultaneous with the initiation of infection threads in root hairs, cells of the root cortex and the pericycle begin to divide, creating a nodule primordium [133,134]. The synchronized occurrence of host cell infection and nodule organogenesis suggests that there is some form of long-range transmission of symbiotic signals [135,136]. In cells of the outer cortex, the nucleus migrates to the center of the host cell, and a cytoplasmic bridge is formed with longitudinal microtubules connecting opposite sides of the cell [134]. The orientation of this cytoplasmic strand sets the path for the subsequent formation of an infection thread. Therefore, it has been termed a pre-infection thread (PIT) [133].

Temperate legumes such as *M. truncatula*, *M. sativa*, *P. sativum*, and *Trifolium* sp. have a permanent meristem at the tip of elongated nodules even after full maturation. These are termed indeterminate nodules (or, more accurately, nodules with indeterminate meristems). In this case, the development and growth of infection threads continues in the post-meristematic tissue of mature nodules, and the bacteria are continuously released into host cells [83]. *Glycine max* (soybean), *Vicia faba* (bean), and *L. japonicus* are tropical legumes that usually form round determinate nodules. Determinate nodules lack a persistent meristem and do not display an obvious developmental gradient [137].

## 5. Propagation of the Infection Thread

**Figure 1:**
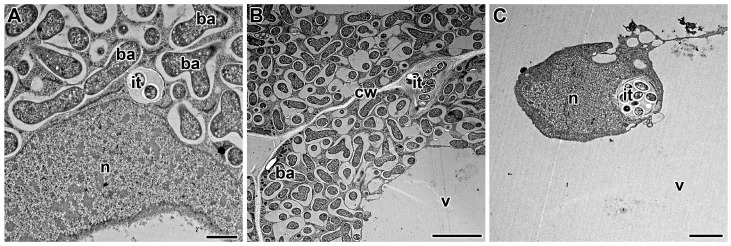
Infection threads in the symbiotic nodules of *Pisum sativum*. (**A**) Intracellular infection thread in the nodules of wild-type line SGE. (**B**) Intercellular infection thread in the nodules of wild-type line SGE. (**C**) Localization of the infection thread in close proximity to the nucleus in the nodules of mutant line SGEFix^–^-1 (*Pssym40-1*). Low-temperature embedding in LR White and transmission electron microscopy. n—nucleus, v—vacuole, cw—cell wall, it—infection thread, ba—bacteroid. Bar (**A**) = 1 µm, (**C**) = 2 µm, (**B**) = 5 µm.

Direct interaction between plant and bacterial cell surfaces plays a critical role in the formation of the infection thread [138]. Morphologically, the infection thread is a tubular ingrowth of the cell wall, surrounded by a plasma membrane and containing a matrix with enclosed bacteria (Figure 1) [17,83]. Rhizobia in the infection thread are in the apoplast and remain physically separated from plant cell cytoplasm [19,83]. The distance between the tip of the infection thread and the rhizobia is constant and does not normally exceed 10 μm [17]. Growth of the infection thread apparently occurs in discrete steps. Rapid cell wall growth at the tip is followed by a phase of bacterial division and sliding growth in the extracellular matrix within the lumen of the thread [19,69,139]. Rhizobia in the infection thread are surrounded by an exopolysaccharide capsule, which may play an important role in facilitating this movement [140,141].

It has also been suggested that the growth and biophysical properties of the infection thread are associated with a transition of the thread matrix from a fluid to a solid-state [17]. Such a transition might result from peroxide-driven cross-linking of tyrosine residues in a composite matrix glycoprotein termed arabinogalactan protein-extensins (AGPEs) [17,142,143,144,145].

Thus, the growth and development of the infection thread are controlled by the host plant with a constant signal exchange between symbionts. Nod factors and low molecular mass exopolysaccharides [146] have been proposed as such signaling molecules. Rhizobia continue to express *nod* genes and synthesize Nod-factor in the infection threads [147,148]. It is thought that different host cell receptor complexes are associated with the initiation of infection, progression of infection thread [86,149,150,151], and even for bacterial release [152].

Extracellular polysaccharides (EPS) can act as signaling molecules that promote the development of infection threads. Indeed, *L. japonicus* was found to have an EPS receptor 3 (LjEPR3) that recognize EPS [153]. Its function is apparently to control the rate of bacterial infection during the growth of an infection thread, both in the epidermis and in subsequent layers of root tissue towards the nodule primordium [154]. Direct binding of EPS to the receptor has been demonstrated [155]. In *M. truncatula LysM domain-containing receptor-like kinase* (*MtLYK10*), an ortholog of *LjERP3* was identified; however, MtLYK10 is not involved in EPS recognition (at least in recognition of succinoglycan), but it is required for infection thread growth [156]. In legumes that form determinate nodules (such as soybean and *L. japonium*), the production of EPS by rhizobia is required for the curling of root hairs, correct formation of infection threads, bacterial release, differentiation of bacteroids, and effective nodulation [25,157,158].

In legumes that form nodules with indeterminate meristems, capsular polysaccharides (KPS) may also play a role in the infection process [159,160]. It appears to stimulate the initiation and development of the infection thread [161]. Bacterial proteins may also play a role in the growth and development of the infection thread. For example, *RosR* encodes a protein belonging to the Ros/MucR family of rhizobial transcriptional regulators, and the mutant *rosR* is impaired in development of infection threads, bacterial release, and differentiation of bacteroids [162].

Many components of the host plant have also been shown to play a role. These include enzymes such as the E3 ubiquitin ligase in *L. japonicus* [87], the putative E3 ubiquitin ligase with the Nodule specific RING finger domain (LjnsRING) in *L. japonicus* [163], the E3 ubiquitin ligase with ‘zinc finger’ type domain, Seven in absentia (MtSINA) in *M. truncatula* [164] and Cystathionine-β-synthase-like1 (MtCBS1) in *M. truncatula* [165]. Recently, using pea gibberellin-deficient and *della*-deficient mutants, it was shown that the phytohormone gibberellin suppresses the formation of infection threads [166], and its amount in infection threads is much lower than in bacteroids [167], on the contrary, phytohormones cytokinins and auxins play an important role in the development and propagation of infection threads, as well as in the release of bacteria from infection droplets [132,168,169,170]. A putative role of ethylene in infection thread maturation was also suggested [171]. In *P. vulgaris*, a small heat shock protein Nodulin 22 (PvNod22) was implicated in nodule development. Its function is associated with the expansion of the infection thread, probably due to the maintenance of protein homeostasis in the ER, since the lack of this protein leads to overloading of ER’s capacity for protein folding [172].

The protein Symbiotic remorin 1 (MtSYMREM1), flotillins MtFLOT2 and MtFLOT4 [88] present in specific microdomains of the infection thread membrane are involved in the regulation of polar growth in the infection thread [173] and possibly interact with MtLYK3 [32]. For *M. truncatula*, it has been shown that vapyrin (MtVPY), putative E3 ligase Lumpy infections (MtLIN), and cytoplasmic exocyst subunit EXO70H4 are part of a symbiosis-specific mechanism required for polar growth of infection threads [21]. Recently, it was shown that LjCerberus stabilizes LjVPY1 and LjVPY2 into trans-Golgi network/early endosome vesicles [174]. In addition, the *Rhizobium-directed polar growth* (*MtRPG*) gene is involved in the spatial subcellular reorganization in *M. truncatula*, encoding a protein belonging to the family of plant-specific proteins with a specific RPG-related proteins (RRP) domain and coiled-coil domain [175]. Infection thread growth also involves the action of small GTPases MtROP6 and MtROP10 [81], and monomeric GTPase RabA2 in *P. vulgaris* [176]. Recently, it was demonstrated that MtROP6 is activated with SPIKE 1 (LjSPK1), a DOCK family guanine nucleotide exchange factor (GEF), and that their interaction is necessary for polar infection thread growth [177]. Transcription factors of the APETALA 2/ethylene-responsive element binding factor (AP2/ERF) family in *L. japonicus* are also important for infection thread growth [178].

The growth and development of the infection thread are accompanied by the movement of the nucleus and the rearrangement of cytoskeletal elements. The nucleus is apparently an active participant in the infection process (Figure 1C). Accompanied by a significant pool of cytoplasm with various organelles, the nucleus moves to the site of contact with the penetrating agent [179], be it pathogen or symbiotic partner. Through this repositioning of the nucleus, signal transduction pathways can perhaps activate gene expression more effectively [180].

Microtubules form a dense cytoplasmic network surrounding the growing infection thread [134,147,181]. This network controls polar growth and serves as a template for the formation of an infection thread. The role of actin filaments is suggested by the presence of a panel of mutants showing the impaired organization of the actin cytoskeleton and impaired polar growth of infection threads. *L. japonius* mutants *Ljarpc1* [116], *121F-specific p53 inducible RNA* (*Ljpir1*) and *nck-associated protein 1* (*Ljnap1*) [76], as well as the *M. truncatula* mutant *Mtrit–1* (ortholog *Ljnap1*) [117] were characterized by a decreased number of infection threads and their disintegration. This led to the formation of ‘empty’ nodules, into which the infection threads did not penetrate [116]. *L. japonicus SCAR-Nodulation* (*LjSCARN*) encodes another component of the SCAR/WAVE complex [182]. *Ljscarn* mutants were blocked at the stage of initiation of infection thread growth. In contrast to the *Ljarpc1*, *Ljnap1*, and *Ljpir1* mutants, in the *Ljscarn* mutants, the organization of actin cytoskeleton was not impaired at the early stages of nodule development. LjSCARN is likely to function at later stages of the actin cytoskeleton reorganization during the development of an infection thread [182].

The endoplasmic reticulum and Golgi apparatus (GA) direct material to the active sites of biosynthesis and remodeling of the infection thread wall [138,183,184,185]. Moreover, the secretion of the components apparently proceeds in two different ways, depending on the stage of cell infection. In a young cell, the route is: ER → Golgi (packaging) → exit → wall; whereas, in a differentiated cell, the route is: ER → vesicle formation → wall. The smooth endoplasmic reticulum tends to be adjacent to the cell wall [183,185]. The GA also has at least two different export pathways, one for pectin-containing vesicles and the other for vesicles containing extracellular membrane and matrix components. Examples include the membrane glycoprotein antigen identified by the antibody MAC207 and the matrix AGPE identified by MAC265 [186]. Vesicles moving out of the Golgi also contain xyloglucan precursors.

### 5.1. Infection Thread Wall

The structure and development of infection threads in the root cortex and in the infected nodule tissue have been extensively studied using microscopic techniques [138]. Having crossed the host cell cytoplasm, the tip of the infection thread fuses with the mother cell wall at the site of exit. Penetration into the adjacent cell involves the local degradation of its cell wall and the re-initiation of a new infection thread [187,188]. This repetitive cell-autonomous process facilitates the overall process of tissue invasion by *Rhizobium* [189]. Infection threads are intracellular and transcellular when they cross plant cells (Figure 1A) and intercellular, when they pass between cells, in which case the plant cell walls effectively serve as the boundary of the intercellular infection thread (Figure 1B).

Intensive genetic studies in various legumes have led to the identification of mutants with defects in the growth and development of infection threads: for *L. japonicus* [87,92,115]; for *M. truncatula* [164,190,191,192]; and for *P. sativum* [166,193,194,195,196]. By using these mutants in combination with monoclonal antibodies and other probes that react with components of the plant-rhizobial interface, it has become possible to analyze surface interactions between symbiotic partners in infection threads (Table 1).

#### 5.1.1. Enzymes Involved in the Growth of the Infection Thread

The growth of an infection thread involves a range of enzymes both for the synthesis of the infection thread wall and for the local degradation of the host cell wall when the infection thread passes through it. There are two possible mechanisms for this process. The first possibility is that bacterial enzymes degrade the cell wall, thus allowing bacteria to penetrate into plant cells [54,106,107]. In *R. etli*, forming nodules on *P. vulgaris*, the gene *HrpW* was isolated, encoding a component of the Type III secretory system. It exhibited pectate lyase activity and may be involved in the degradation of the cell wall during infection thread development [224].

A second possible mechanism for cell wall degradation involves plant enzymes. Cell wall degrading enzymes are produced in nodule cells in response to rhizobial infection and are possibly induced by Nod factors [188]. In *M. sativa,* the polygalacturonase gene (*MsPG3*) is specifically expressed during symbiosis [110]. Another degradative enzyme is Nodulation pectate lyase (LjNPL) [112]. The identified pectate lyase is probably only one of several proteins associated with the initiation and growth of an infection thread. In addition, PMEs are apparently involved in the modification of the pectin matrix of the infection thread wall [106]. Thus, it was described in nodules on the adventitious roots of *Sesbania rostrata* Bremek. and Oberm. after inoculation with *Azorhizobium caulinodans* [225]. In *M. truncatula,* after inoculation with *S. meliloti*, the expression of symbiotic *Pectin methylesterase* (*MtPER*) was identified [226]. In pea nodules, the participation of endo-β-1,4-glucanases in the maturation of the infection thread and cell walls was shown [217].

#### 5.1.2. Polysaccharides and Proteins of the Infection Thread Wall

The infection thread wall is an extension of the host cell wall. It includes esterified and de- esterified homogalacturonan (HG), substituted pectins, xyloglucans and cellulose microfibrils [15,197,207] as well as extensin. In the nodules of *P. vulgaris* and *M. truncatula*, the presence of proline- and hydroxyproline-rich glycoproteins has been demonstrated [214,227]. In addition, rhizobial infection has been shown to modulate the gene expression for extensins and expansins, both structural proteins of the cell wall [228,229,230]. In *M. truncatula*, in response to infection, genes of early nodulins, such as *Early nodulin 5 (ENOD5)*, *Early nodulin 12 (ENOD12)*, *Early nodulin 16/20 (ENOD16/20)*, which are proline- and hydroxyproline-rich proteins, begin to be expressed in cells with actively growing infection threads [231]. The involvement of ENOD5 at the late stages of infection was demonstrated using a large set of symbiotic pea mutants blocked at different stages of infection [232].

**Figure 2:**
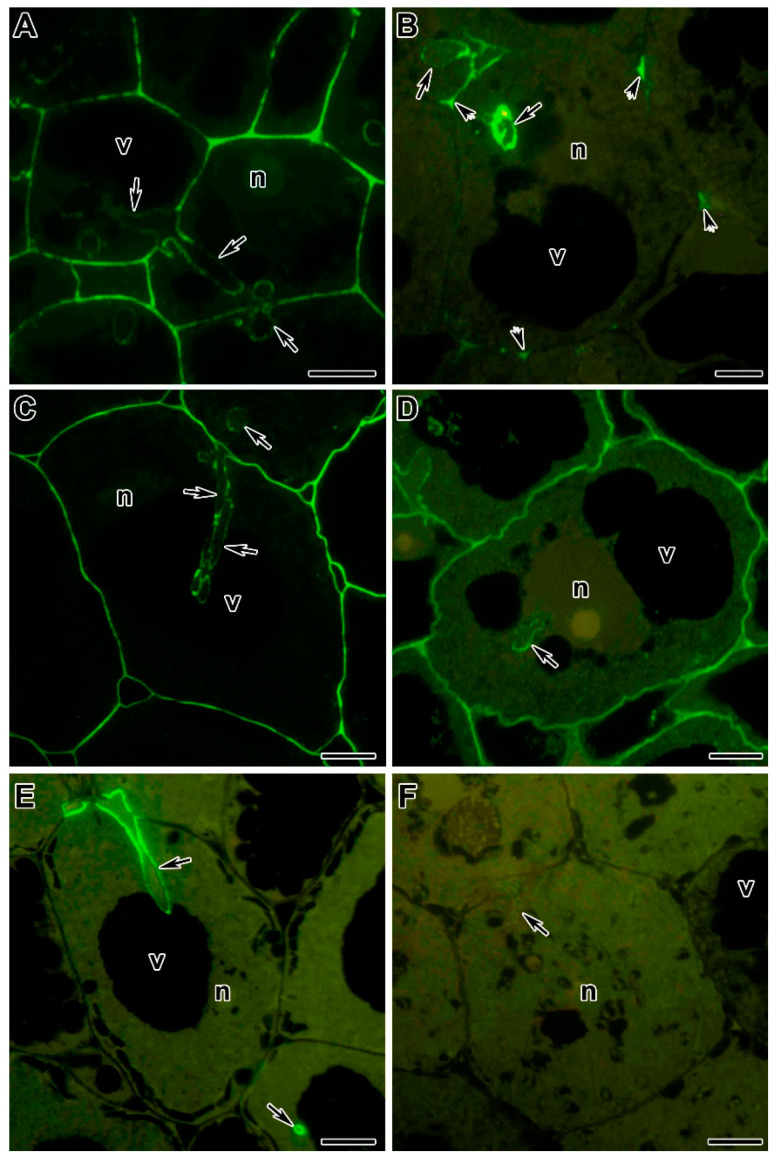
Pectins in the infection thread walls in the symbiotic nodules of *Pisum sativum* and *Medicago truncatula*. (**A**,**B**) Low methyl-esterified homogalacturonan (HG) labelled with JIM5. (**C**,**D**) High methyl-esterified HG labelled with JIM7. (**E**,**F**) (1→4)-β-D-galactan sidechain of rhamnogalacturonan I labelled with LM5. (**A**,**C**,**E**) Nodules of the wild-type line SGE of *P. sativum*. (**B**,**D**,**F**) Nodules of the wild-type line A-17 of *M. truncatula*. n—nucleus, v—vacuole. Arrows indicate infection threads, arrowheads indicate ‘three-way’ junctions. Low temperature embedding in LR White, semi-thin sections (0.5 µm), fluorescent immunolocalization. Bars = 5 µm.

The pectins of the infection thread wall are diverse [233], and their detailed localization has been examined by immunocytochemical microscopy (Figure 2). The distribution of HG was studied in various legumes. Infection thread walls were shown to have a high content of low methyl-esterified HG in *P. sativum*, *M. truncatula*, *Vicia hirsuta* (L.) Gray, and *P. vulgaris*, (Figure 2A,B) [186,197,203,216]. There was also evidence for high methyl-esterified HG (Figure 2C,D) [197,203]. When vesicular transport was impaired in *M. truncatula* and *G. max* in nodules with partially silenced *VAMP721d* and *VAMP721e*, large clusters of bacteria were found immersed in a matrix of high and low methyl-esterified HG, surrounded by a membrane [205,234]. At the same time, GmVAMP721d partially co-localized with pectate lyase, and abnormal endocytosis of low methyl-esterified HG was observed [205].

Mutants of *P. sativum* and *M. truncatula* with defects in infection thread development have been investigated for possible changes in the composition of HG in infection thread walls. Mutants in the gene *PsSym33* and the orthologous gene *MtIPD3*, characterized by ‘locked’ infection threads, showed a strong accumulation of low methyl-esterified HG in the infection thread walls [201,203]. By contrast, in symbiotically-defective mutants without abnormalities in infection thread development, the distribution of low methyl-esterified HG did not differ from that in wild-type plants [203].

Rhamnogalacturonan I (RG-I) consists of alternating (1,2)-linked α-L-rhamnose residues and (1,4)-linked α-D-galacturonic acid residues. Its role in the development of the legume-rhizobial symbiosis has not been studied until recently [203]. Using the LM5 antibody raised against galactan side chain epitope, it was shown to be present in infection thread walls in *P. sativum* nodules but was not detected *M. truncatula* nodules (Figure 2E,F). At the same time, in the pea mutant SGEFix^−^-2 (*Pssym33-3*), the LM5 epitope was absent from the walls of some infection threads [203].

Rhamnogalacturonan II (RG-II) is structurally the most complex but also the most conserved pectin polysaccharide. RG-II macromolecules are self-conjugated as dimers through a diester bond with boron [235]. When studying the effect of boron on nodulation, the localization of RG-II at the interface between the plasma membrane and the cell wall was shown [199]. However, in plants deficient in boron, RG-II was evenly distributed over the entire thickness of the cell wall [199]. Later, it was shown that RG-II can form a complex with AGPEs and was localized in the infection thread matrix [200,206].

### 5.2. Infection Thread Matrix

Production and secretion of plant extracellular matrix material are stimulated in response to rhizobial infection. Most of these compounds accumulate in the lumen of the infection thread. According to some estimates, for *P. sativum* and other legumes that form indeterminate nodules, the volume of matrix material in the infection thread lumen is approximately five times the volume of rhizobial cells [236].

The main components of the matrix within the infection thread lumen are plant glycoproteins, basically similar to those of the extracellular matrix. AGPs are found widely in plants, but legume nodules contain a tissue-specific set of AGPs. This class of hydroxyproline-rich glycoproteins is found in infected tissues of symbiotic nodules of legumes, in actinorhizal symbiosis, and in arbuscular mycorrhiza [17,237,238]. AGPs apparently play a significant role in the infection process, most likely in the symbiotic interface [237,238,239].

Legumes are apparently unique in their ability to synthesize a complex copolymer that contains alternating AGP and extensin motifs [17,141,144,238]. AGPE molecules appear to combine the biophysical properties of soluble gums (characteristic of AGPs) with the more structural properties of extensins (which usually serve to strengthen plant cell walls). The high content of tyrosine residues in AGPEs suggests the possibility of cross-linking of these molecules with H_2_O_2_ and Prx-III, as with the extensin network in many plant cells [144,145]. This cross-linking may serve to regulate the growth of the infection thread itself [17,142,206,240].

AGPE, with an apparent molecular weight of 95 kDa or larger, was identified in extracts from symbiotic nodules using three monoclonal antibodies MAC204, MAC236, and MAC265. These antibodies apparently recognize different epitopes on the same group of glycoprotein macromolecules [186,212]. The two epitopes recognized by MAC236 and MAC265 were mutually exclusive as seen from isoelectric focusing, while MAC204 recognized a periodate-sensitive epitope common to both the acidic and neutral forms. AGPEs were immunolocalized to the infection thread matrix. Although its abundance is increased in nodule tissue extracts, it is not a classic nodulin [241]. It is accumulated in uninfected root tissue, in particular in intercellular spaces bounded by three or more cells [186,207,208,212,242]. In addition, AGPE recognized by MAC265 was also found in the intercellular spaces of pseudo-nodules induced by the LPS-defective mutant of *R. leguminosarum* [208].

**Figure 3:**
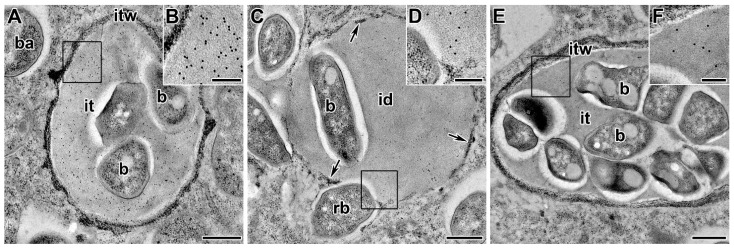
Arabinogalactan protein-extensins (AGPEs) in the infection thread matrix in the symbiotic nodules of *Pisum sativum*. (**A**) AGPE labeled with MAC265. (**B**) High magnification of the boxed area in (**A**). (**C**) AGPE labeled with MAC204. (**D**) High magnification of the boxed area in (**C**). (**E**) AGPE labeled with MAC236. (**F**) High magnification of the boxed area in (**E**). Low-temperature embedding in LR White, immunogold localization, transmission electron microscopy. id—infection droplet, it—infection thread, itw—infection thread wall, b—bacterium, rb—released bacterium, ba—bacteroid. Arrows indicate remnants of the infection thread wall. Bars = 500 nm.

The exact function of AGPEs in the growth of infection threads is unknown. Immunocytological analysis showed that AGPE recognized by MAC265, is localized in the infection pocket of curled root hairs, in young infection threads in the infection zone, and in mature infection threads in the nitrogen fixation zone of symbiotic nodules of *P. sativum* (Figure 3A), *V. sativa*, *P. vulgaris* [141,186,197,210,211]. A similar distribution of AGPE epitopes is observed using MAC204 and MAC236 in *P. sativum* (Figure 3C–F) [186,213]. However, in pea mutants with abnormalities in infection thread development and evidence of bacterial degradation in the lumen, the MAC265 and MAC236 epitopes were observed in excessive amounts in the intercellular spaces of the infected nodule tissue. There was also accumulation of the MAC204 epitope in the cell wall, perhaps as a result of abnormal infection thread development [211,213]. These studies indicate that the nature of AGPE macromolecules may be subject to change during the infection process [213].

Borate is an essential micronutrient for legume nodule development. An effect of boron deficiency on the distribution of matrix AGPE in *P. sativum* nodules has been demonstrated [209]. In *P. sativum* plants, the RG-II complex with boron and AGPE was observed in the infection thread matrix, while the rhizobial cells were separated from the matrix by an exopolysaccharide capsule. In nodules of plants deficient in boron, the complex of AGPE with RG-II was strongly associated with the surface of rhizobia in the infection thread lumen [206].

### 5.3. ROS and NO

**Figure 4:**
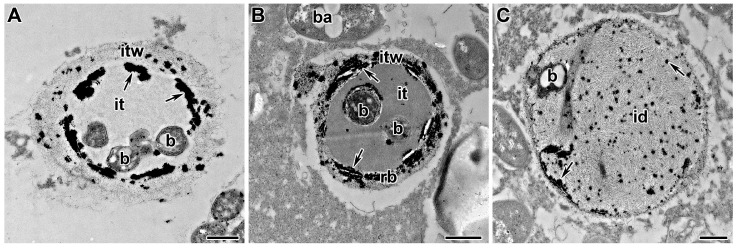
Sequential steps of hardening of the infection thread wall and matrix during development and growth in the wild-type nodules of *Pisum sativum*. (**A**) Localization of H_2_O_2_ in the inner side of infection thread wall. Incomplete hardening of the wall of the infection thread makes it possible to form an infection droplet and release the *Rhizobium* into the plant cell. (**B**) Localization of H_2_O_2_ across the entire infection thread wall thickness. Complete hardening of the infection thread wall prevents the formation of an infection droplet and the release of bacteria. (**C**) Localization of H_2_O_2_ inside infection droplet matrix. Hardening of the infection droplet prevents the growth and division of bacteria inside the lumen. Cytochemical localization of H_2_O_2_ as electron-dense precipitate formed in the presence of cerium chloride. id—infection droplet, it—infection thread, itw—infection thread wall, b—bacterium. Arrows indicate electron-dense precipitates. Bars = 500 nm.

Reactive oxygen species (ROS) are produced during infection by rhizobia [243]. Rbohs have been identified and characterized in legume genomes [123,244,245]. It has been shown that RbohA and RbohB can play a key role in the successful colonization of rhizobia and the correct growth and shape of infection threads, apparently because they stimulate ROS production [244,245]. Prx-III, as well as rhizobial catalases [222], affect the rigidity of the infection thread wall and matrix [142]. During infection, the production of superoxide anion (O_2_^-^) and H_2_O_2_ were localized in infection threads and infected cells [219,221]. At the same time, it is possible to trace the accumulation of H_2_O_2_, first on the inner surface of the infection thread wall (Figure 4A), then throughout the entire thickness (Figure 4B), and then inside the infection thread matrix (Figure 4C), possibly promoting its hardening as a result of crosslinking of tyrosine residues of AGPE molecules [17,144]. Thus, it was suggested that the role of H_2_O_2_ during extension of the infection thread is associated with the rigidity of the infection thread [125,141,145,246] or with the signaling role of H_2_O_2_ for the regulation of symbiotic function [125].

In addition to Rbohs, there are several other potential sources of reactive oxygen species. Diamine oxidase (DAO) is an important source of hydrogen peroxide in intercellular spaces in legume tissues, both in intact plants and in plants exposed to various stresses [142,220]. Peroxide distribution and DAO activity in nodules have been demonstrated in plant cell walls, intercellular spaces, and infection threads. Similarly, in symbiotic nodules of *P. sativum* the localization of lipoxygenase (LOX) in the infection thread matrix was demonstrated [218]. This enzyme is involved in lipid peroxidation, and its accumulation may indicate a hypersensitivity reaction that develops in response to rhizobial infection. However, EPS-I of rhizobia can reduce the effects of H_2_O_2_ on bacteria [247]. Nitric oxide production was also observed along growing infection threads and in nodule primordia [129].

### 5.4. Defense Reactions

Bacterial colonization triggers non-specific plant defense responses [248,249]. Mutants of *S. meliloti*, *R. leguminosarum* bv. *trifolii*, *R. leguminosarum* bv. *viciae*, *B. japonicum*, and *A. caulinodans*, unable to produce EPS, induce defense reactions in their respective hosts *M. sativa*, *Trifolium* sp., *V. sativa* sp. *nigra*, *G. max* and *S. rostrata* [42,250]. In such cases, later colonization and histochemical changes in the cortical cell walls of the pseudo-nodule are observed. They are abnormally thickened, encrusted with autofluorescent phenolic compounds, and contain callose [251,252]. In addition, phytoalexins (glyceolin) accumulate in pseudo-nodules, peroxidase activity is increased, as well as the levels of phenylalanine ammonium lyase, 3-O-methylesterase, and isoflavone reductase transcripts, which indicates the occurrence of typical defense reactions [248]. It is likely that EPS produced at an early stage of infection is necessary as a diffusion barrier protecting bacteria from toxic H_2_O_2_ generated in plant cells [42,253,254].

Allelic mutants of *lateral root organ-defective* (*latd*) [255] and *numerous infections and polyphenolics* (*nip*) were identified in *M. truncatula* with defects in the architecture of the root and nodules [191,192]. *NIP/LATD* gene encodes a putative nitrate transporter [256]. In *latd* and *nip* mutants, the accumulation of polyphenolic compounds and abortion of infection were observed either at the stage of propagation of the infection thread or during the release of rhizobia into the cytoplasm of plant cells [256]. A similar phenotype was also observed for TE7 mutant [190] in the *MtIPD3* gene [257].

**Figure 5:**
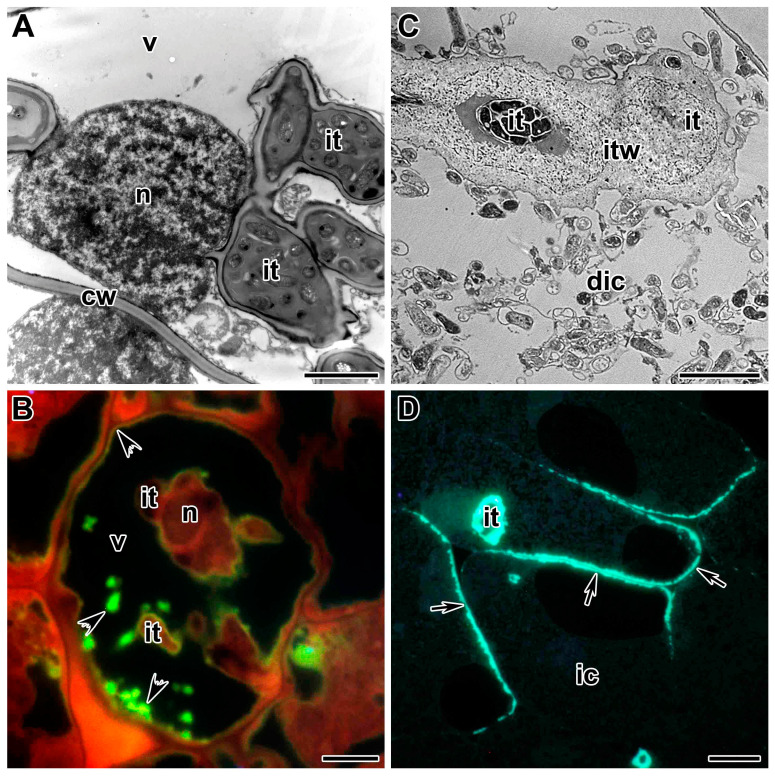
Defense reactions in the nodules of symbiotic *Pisum sativum* mutants. (**A**) Transmission electron micrograph of abnormal infection threads in the nodule of mutant SGEFix^–^-2 (*Pssym33-3*). (**B**) Suberin depositions in the infection thread walls and around vacuole in the nodule of mutant SGEFix^–^-2 (*Pssym33-3*), which is characterized with ‘locked’ infection threads. Neutral red staining for detection of suberin. (**C**) Transmission electron micrograph of an abnormal infection thread in the nodule of mutant RisFixV (*Pssym42*). (**D**) Callose (β-1,3-glucan) depositions in the infection thread wall and cell wall of infected cells in the nodule of mutant RisFixV (*Pssym42*), which is characterized with abnormal infection threads and early senescence of symbiotic structures. Callose depositions detected by staining with Aniline blue. ic—infected cell, dic—degenerated infected cell, n—nucleus, v—vacuole, cw—cell wall, it—infection thread, itw—infection thread wall. Arrows indicate callose depositions in cell walls of infected cells, arrowheads indicate suberin depositions in the vacuole. Bars (**A**) = 2 µm, (**B**–**D**) = 5 µm.

Later, it was found that, for pea mutants in the *Pssym33* gene (ortholog to MtIPD3), the deposition of suberin was observed in the infection thread walls (Figure 5A,B) [201]. In addition, in this mutant, suberin was present in the cell walls of colonized cells. It was also found in the infection thread walls and around the vacuole of infected cells (Figure 5B) in the nodules of the weak allele SGEFix^–^-2 (*Pssym33-3*) [204]. In some infection threads of this pea mutant, an electron-dense matrix was also observed when labeled with the LM5 antibody, recognizing the galactan side chain of RG-I [204]. There was also deposition of de-esterified HG in the infection thread walls and an increase in the expression level of a gene encoding peroxidase 7RA84 [201]. The deposition of cell wall material inside the vacuole and the formation of a pectin gel in the infection thread matrix is another manifestation of the host plant’s defense reaction and the perception of rhizobia as pathogens.

The deposition of callose (β-1,3-glucan) in plant cell walls is an important aspect of many processes associated with developmental physiology, pathogenesis, or stress. In the *P. sativum* symbiotically-defective mutant RisFixV (*Pssym42*), callose deposition (Figure 5C,D) was associated with *Rhizobium* infection as part of the defense response. Another striking feature of RisFixV (*Pssym42*) was the encapsulation of ineffective bacteroids with de-esterified HG, as detected by JIM5 immunolabelling. This mutant demonstrates unique defense reactions for symbiotic mutants [201].

## 6. Release of Bacteria from Infection Threads

The process of tissue and cellular infection is accompanied by the differentiation of plant cells originating from the apical meristem. The differentiation of host cells in the nodule cortex is associated with the release of bacteria into the plant cell as organelle-like structures termed symbiosomes, which are still bounded by a plant membrane that is structurally equivalent to the plasma membrane [258].

The exact mechanism triggering the transition of rhizobia from the extracellular space (apoplast) to intracellular existence within organelle-like symbiosomes surrounded by a plant membrane is not yet known, but it is associated with further remodeling of the host cell wall and cell membrane. In indeterminate nodules of temperate legumes, the release of rhizobia occurs from infection droplets that lack a covering of cell wall material [17,236,259]. These droplets are confined by a membrane that is an extension of the plant plasma membrane [138]. They contain a matrix similar in composition to the matrix of the infection thread. This includes AGPEs, which is recognized by the monoclonal antibodies MAC265, MAC236, and MAC204 (Figure 3B) [197,211,213].

Sometimes, for example, in *Phaseolus* sp., bacterial release occurs at the tips of short intracellular infection threads [260]. A study of the tips of infection threads in the cytoplasm of host cells of *Lupinus angustifolius* L. showed that rhizobia bud off from infection threads and are enclosed in membranes of plant origin [261]. The released bacterial cells eventually stop dividing and differentiate into an endosymbiotic, nitrogen-fixing form (bacteroids). In nodules of some legumes, differentiation of bacteroids occurs as a result of the action of antimicrobial nodule cysteine-rich (NCR) peptides [262].

Presumably, the release of bacteria occurs due to the absence of a cell wall in the infection droplet and the possibility of close contact between the plasma membranes of the plant and rhizobia [17,197,263]. The physical interaction of isolated symbiosomal and bacterial membranes has been demonstrated in vitro [264]; moreover, when rhizobia enter the cytoplasm of plant cells, they lose their exopolysaccharide capsule (Figure 3B) [140]. Many components associated with symbiosomal and plasma membranes may play a direct role in surface interactions with rhizobia [265].

On the part of rhizobia, the gene *BacA* controls the modification of the bacterial cell wall, including the development of Lipid -A derivatives with long-chain fatty acids [266]. During bacteroid development, modifications to Lipid-A and O-antigen sidechains cause rhizobial LPSs to become more hydrophobic [267,268], which may facilitate interaction with plant membrane glycoproteins [239]. Evidence that the O-antigen of LPS plays an important role in this process comes from the observation that rhizobial mutants defective in its production are not able to release from infection droplets [269].

## 7. Nodule Senescence and Release of Bacteria

Senescence of nodule tissue is the final stage of symbiosis. There is autolysis of infected cells, and the nutrients stored in the nodules are recycled by the host plant [270]. Proximal to the senescence zone, a network of intercellular infection threads develops among degenerating cells [271,272]. Rhizobia multiply as saprophytic organisms, thus enhancing the population of bacteria that can be released into the soil [271]. A similar infection network is observed during ineffective symbiotic associations leading to early senescence [273,274]. Infection threads in the saprophytic zone sometimes form large infection droplets filled with a matrix of unknown nature [272].

## 8. Environmental Influences

The extent of nodulation and the efficiency of symbiotic nitrogen fixation can be influenced by environmental conditions, such as temperature, humidity, aeration, pH, salinity, soil structure, imbalance of nutrients in the soil (for example, nitrogen, phosphorus, calcium, boron, potassium, and magnesium). Other factors affecting nodulation include diseases and insects, as well as anthropogenic influences in the form of fertilization, soil and water pollution with pesticides, chemicals, and heavy metal ions [275,276]. Environmental stresses often have diverse effects: For example, pesticides can improve plant viability by suppressing pests, or they can have a direct toxic effect on nodule metabolism [277]. The stress sensitivity of the legume-rhizobial symbiosis has been extensively studied, although the growth dynamics of an infection thread under abiotic stress are not easy to analyze. It should be noted that stress sensitivity can sometimes be reduced by inoculation with strains of rhizobia resistant to various stresses [278,279,280].

## 9. Conclusions and Perspectives

**Figure 6:**
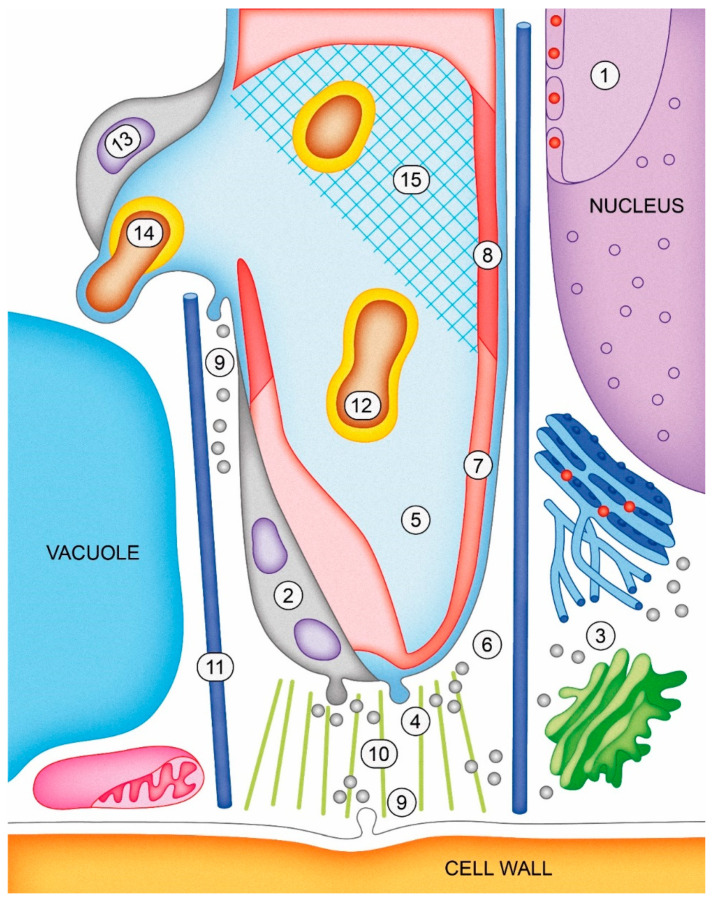
Structure and development of the legume-rhizobial symbiotic interface in infection threads (see text for details). (**1**) Transcription factors modify the course of host cell development. (**2**) Plant proteins and glycoproteins are localized in the microdomains of the plasma membrane of the infection thread. (**3** and **4**) Vesicles derived from the endoplasmic reticulum and Golgi apparatus are directed to the plasma membrane at the tip of the infection thread, releasing their contents into the wall and extracellular matrix. This secretory mechanism is the driving force for the growth of the infection thread, coupled with the growth of bacterial cells within the luminal matrix. (**5**) Glycoproteins, proteins, and polysaccharides influence the biophysical properties of the infection thread wall and luminal matrix. (**6**) Cell wall proteins and polysaccharides are transferred in vesicles from the Golgi apparatus. (**7**) In the nascent wall of the infection thread, there is a layer of α-cellulose, and HG is predominantly in the highly methyl-esterified form. (**8**) The mature wall of the infection thread contains cellulose, xyloglucan, and HG with decreasing degrees of methyl-esterification. This creates a more rigid structure reinforced by bridging with Ca^2+^ ions. Also present is RG-I and RG-II (in the form of dimers with the borate ion), extensins, AGPs, and expansins. (**9**) During the development of the infection droplet, enzymes are involved in the modification and degradation of the cell wall. (**10**) The actin cytoskeleton is involved in the organization of polar growth. (**11**) The microtubular cytoskeleton forms a tunnel for orientation. (**12**) Polysaccharides and proteins of the bacterial cell wall and capsule play an important role in the progression of the infection thread. (**13**) The symbiosome membrane contains a new range of proteins associated with nitrogen-fixing endosymbiosis. (**14**) Within the symbiosomes, rhizobial cells lose their exopolysaccharide capsule, and the structure of LPS is modified. (**15**) H_2_O_2_ plays a role in cross-linking of AGPEs and hardening of the infection thread matrix. HG, homogalacturonan; RG-I, rhamnogalacturonan I; RG-II, rhamnogalacturonan II. Green lines are actin microfilaments; blue lines are microtubules; 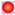
—Ca^2+^; light blue extracellular matrix is fluid; shaded blue matrix is solid; pink cell wall is newly synthesized; red cell wall is mature or modified. Objects are not scaled.

Most plants have two types of tip-growing cells: Pollen tubes and root hairs. Infection threads represent a third type of tip growth for the deposition of cell wall material. The infection thread is an inwardly growing tube in which polar (apical) growth is topologically inverted relative to the tip growth of root hairs or pollen tubes. The following model (Figure 6) summarizes basic concepts of infection thread growth.

The initiation and growth of the infection thread are the consequence of signal exchange with the infecting *Rhizobium* bacteria and altered transcriptional activity in the host cell nucleus. Among other things, this leads to the synthesis and deposition of new proteins in the microdomains of the plasma membrane of the infection thread. Vesicles derived from the ER and GA fuse with the plasma membrane at the tip of the infection thread, releasing their contents into the wall and extracellular matrix. This process is apparently controlled by a range of proteins, including: PI3K, GmVAMP721d, EXO7OH4, PvNod22. Targeted secretion from vesicles (together with the growth and division of bacteria within the luminal matrix) is the driving force behind the growth of the infection thread. Glycoproteins, proteins, and polysaccharide components of the infection thread matrix include the following: AGPEs, HRGPs, ENOD2/11, LOX, DAO and RG-II. In addition, H_2_O_2_ probably plays a role in cross-linking AGPEs and changing the biophysical properties of the infection thread matrix. Vesicles derived from the GA also contain cellulose synthases, xyloglucan and pectins (HG, RG-I, and RG-II). HG is synthesized in a highly esterified methyl form and may be transported along with PME/PMEI complexes (pectin methylesterase/pectin methylesterase inhibitor). All these components and other cell wall remodeling enzymes are released into the apoplast.

In the nascent wall of the infection thread, cellulose synthases are incorporated into the membrane and deposit the crystalline cellulose. At this stage, highly methyl-esterified HG is the major component of the infection thread wall. At its mature stage, the main polysaccharide components are: cellulose, xyloglucan, and HG with varying degrees of methyl-esterification. HG having a low level of methyl-esterification binds with Ca^2+^ ions, thus increasing its rigidity. RG-I is present in the infection thread wall, and RG-II is also present in the form of dimers with the borate ion. The wall also contains extensins, AGPs, and expansins. Additionally, callose and phenolic compounds such as suberin can accumulate as part of defense reactions in response to an ineffective symbiosis.

Many enzymes are involved in the modification and degradation of the cell wall during the growth of an infection thread and the formation of an infection droplet. These include: MsPG3, LjNPL, MtPER, and endo-β-1,4-glucanases. Polar growth of the infection thread is mediated by the actin cytoskeleton, and the alignment of microtubules creates a constraining tunnel for infection thread growth. Within the lumen, bacterial polysaccharides play an important role: EPS, KPS, LPS, cyclic β-glucan, RosR, and Rhizobia-induced peroxidases (Rip1-10).

Following the release of bacterial cells from the infection droplet, several plant proteins are associated with the symbiosomal membrane. These include: Lectin-like glycoprotein (PsNLEC1), synaptotagmin (MtSyt1/2/3), syntaxin (MtSYP132), inositol-containing phospholipid (JIM18 antigen), and AGP with a GPI anchor (JIM1 antigen). Within the symbiosome compartment, rhizobial cells differentiate into nitrogen-fixing bacteroids: They lose their exopolysaccharide capsule, and the structure Lipid-A and O-antigen groups of LPS becomes modified, partly as a result of the action of the protein BacA.

From an evolutionary perspective, the nature of the legume-rhizobial symbiotic interface has become progressively more intimate and complex. On the one hand, it incorporates novel aspects of cellular morphogenesis, in particular the infection thread and the symbiosome compartments. On the other hand, there is a precise system for suppression of host defense responses. As with the other forms of tip-growth observed in root hair cells and pollen tubes, growth of the infection thread is due to the targeted deposition of cell wall and cell membrane material at the apex. However, there is an important distinction. Whereas the extension of root hairs and pollen tubes is driven by cell turgor, the driving force behind the growth of an infection thread is the synthesis and directed secretion of the extracellular matrix material into the lumen of the infection thread and the division of bacteria inside. Polar growth of the infection thread requires a high degree of coordination between many cellular and extracellular processes, including calcium dynamics, apoplastic reactive oxygen species, the cytoskeleton, and vesicular transport.

The cell wall that surrounds the infection thread is a dynamic structure that performs both structural and defense functions. While the localization and distribution of the main pectins of the infection thread wall have been recently studied [203], the role of other components of the cell wall, including numerous proteins, remains poorly understood. Many questions arise. How are internal and external processes coordinated during the growth of infection threads? What controls the progressive change in composition of the infection thread wall? What is the role of cell wall proteins? What is the role of bacterial components? How are the cell wall and matrix of the infection thread modified in response to abiotic stresses?

Experiments designed to investigate these questions will provide new insights into how an infection thread grows. Furthermore, these studies will help to elucidate the more general patterns of plant cell wall development during growth and differentiation. New probes targeting cell wall components will permit a more detailed analysis of the biochemistry and biomechanics of the cell wall of the infection thread. A major problem in studying the infection thread is that it is a dynamic and continually changing system. Its structure at an early stage in a root hair or root cortical cell may be very different from that in a host cell embedded deep in the tissue of a maturing nodule. These differences could affect the remodelling of the cell wall (Figure 6). The challenge for further research will be to use genetics, genomics, and cytological studies to integrate the many parameters involved in the development of infection threads, ranging from signaling and protein transport to deposition and remodeling of the plant cell wall.

One of the intriguing questions regarding the infection of legume tissues by *Rhizobium* is the relationship between intercellular and intracellular modes of infection. An interesting model has recently been developed based on interaction of *L. japonicus* with different strains capable of infecting the host plant either via intracellular or via intercellular modes [281]. Furthermore, the presence of infection threads in root hairs during actinorhizal symbiosis [151] and the existence of common genes controlling *Rhizobium* infection and endomycorrhizal symbiosis [282,283] clearly indicate the early origin of infection threads during the course of the evolution of plants.

## Figures and Tables

**Table 1:** List of molecular probes (antibody, cytochemical reagent, and enzyme) used to detect infection thread cell wall and matrix components.

Probe ^a^	Component ^b^	Epitope Recognized ^c^	References
Antibody			
JIM5	low methyl-esterified HG	α-MeGalA_(2)_-(1→4)-α-GalA_(3)_- (1→4)-α-MeGalA	[186,197,198,199,200,201,202,203,204]
JIM7	high methyl-esterified HG	α-GalA-(1→4)-α- MeGalA_(4)_-(1→4)-α-GalA	[197,202,203,204]
LM19	low methyl-esterified HG	α-GalA-(1→4)_(4)_	[205]
LM20	high methyl-esterified HG	α-MeGalA-(1→4)_(4)_	[205]
2F4	calcium cross-linked HG	dimer of α-MeGalA-(1→4)_(9)_ and Ca^2+^_(5)_	[202]
LM5	(1→4)-β-D-galactan (RG-I)	β-Gal-(1→4)_(3)_	[203,204]
anti-RG- II	monomeric and dimeric RG-II	unknown	[199,200,205,206]
anti- XyG	XyG	unknown	[197,207]
anti-callose	callose ((1→3)-β-D-glucan)	β-Glc-(1→3)_(5)_	[205]
MAC265	95kDa AGPE	unknown	[141,186,197,205,208,209,210,211]
MAC204	95kDa AGPE	unknown	[186,212,213]
MAC236	95kDa AGPE	unknown	[186,213]
JIM13	AGP	unknown	[208]
anti-HRGP	hydroxyproline-rich glycoproteins (HPGPs)	unknown	[214]
anti-ENOD2	early nodulin2/hydroxyproline-rich glycoproteins (ENOD2/HPGPs)	unknown	[215,216]
anti-VAMP721d/VAMP721e	vesicle-associated membrane proteins (VAMPs)	peptide QKLPSTNNKFTYNC	[205]
anti- EGL1	endo-β-1,4-glucanase	peptide CYFPKRIHHRGSSLP	[217]
anti-LOX	lipoxygenase (LOX)	unknown	[218]
anti-SOD	superoxide dismutase (SOD)	unknown	[219]
anti-DAO	diamine oxidase (DAO)	unknown	[220]
Cytochemical reagent			
chlor-zinc-iodide	cellulose	na	[15]
I_2_KI (I_2_, KI, H_2_SO_4_)	cellulose	na	[15]
cerium chloride (CeCl_4_)	H_2_O_2_	na	[219,220,221,222,223]
ruthenium red	unesterified HG	na	[202,204]
aniline blue	callose ((1→3)-β-D-glucan)	na	[201,204]
neutral red	suberin	na	[201,204]
I_2_KI (I_2_, KI, H_2_SO_4_)	suberin	na	[204]
Protein			
CBH-I	cellulose	na	[197]

^a^ JIM, John Innes Monoclonal, LM, Leeds Monoclonal, MAC, Monoclonal Antibody Centre (Babraham), RG-II. Rhamnogalacturonan-II, CBH-I. Cellobiohydrolase-I. ^b^ XyG. Xyloglucan, HG. Homogalacturonan, RG-I. Rhamnogalacturonan-I. ^c^ Gal, galactose, GalA, galacturonic acid, MeGalA, 6-O-methyl-galacturonate, na. not applicable.
